# Minimum Dietary Diversity Scores for Women Indicate Micronutrient Adequacy and Food Insecurity Status in South African Towns

**DOI:** 10.3390/nu9080812

**Published:** 2017-07-28

**Authors:** Gamuchirai Chakona, Charlie Shackleton

**Affiliations:** Department of Environmental Science, Rhodes University, Grahamstown 6140, South Africa; c.shackleton@ru.ac.za

**Keywords:** dietary intake, dietary diversity, food prices, agriculture, wild foods, rural-urban continuum, reproductive age, food security

## Abstract

The lack of dietary diversity is a severe problem experienced by most poor households globally. In particular; women of reproductive age (WRA) are at high risk of inadequate intake of micronutrients resulting from diets dominated by starchy staples. The present study considered the diets, dietary diversity, and food security of women aged 15–49 years along the rural-urban continuum in three South African towns situated along an agro-ecological gradient. A 48 h dietary recall was conducted across two seasons with 554 women from rural, peri-urban, and urban locations of Richards Bay, Dundee, and Harrismith. Minimum Dietary Diversity for WRA (MDD-W) were calculated and a dichotomous indicator based on a set of ten food groups was used to determine if women had consumed at least five food groups the previous 48 h to achieve minimum dietary intake for women. The mean (±sd) MDD-W for Richards Bay (3.78 ± 0.07) was significantly higher than at Dundee (3.21 ± 0.08) and Harrismith (3.36 ± 0.07). Food security and MDD-W were significantly higher in urban locations than in peri-urban or rural ones. There was lower dependence on food purchasing in Richards Bay compared to Dundee and Harrismith. The majority of women in Richards Bay practiced subsistence agriculture, produced a surplus for sale, and collected wild foods which improved dietary intake and food security. The peri-urban populations had limited dietary intake and were more food insecure because of high levels of poverty, unemployment, and lack of land. Peri-urban dwellers are therefore more sensitive to changes in incomes and food prices because they lack safety nets to absorb income or price shocks as they purchase more, rather than growing their own food. This compromises dietary diversity as they have limited access to diverse foods.

## 1. Introduction

The burden of food insecurity and malnutrition remains a global challenge [1,2], especially in sub-Saharan Africa, where the number of hungry and undernourished people has increased between 2014–2016 [2]. Despite the overall progress to reduce global food insecurity and chronic undernourishment, sub-Saharan Africa remains the most food-insecure region in the world with close to 223 million people undernourished [2,3]. The limited decline in undernutrition rates being linked to low levels of household food security [2,3,4]. This has made it difficult to achieve the first Millennium Development Goal (MDG) which sought to “eradicate extreme poverty and hunger” by 2015, and to halve the proportion of those suffering from hunger [5] as more than one in four people are still undernourished [3].

The prolonged problem of energy deficiencies has made it a continued focus of global efforts to deal with the issue of malnutrition [6]. Food and agricultural productivity has been increased through extensification and intensification to meet the needs of all people in the world yet this did not assure food security for all [7]. Some poorer societies and communities lack access to sufficient quantity or quality food as close to one billion people are facing challenges of not having enough food and two billion are suffering from micronutrient deficiencies [8]. When food is available, many low-income households consume monotonous diets which are of low quality, cereal based, and lacking in vegetables, fruit, and animal-source foods, thereby increasing the risk of micronutrient deficiencies which is already high in some resource-poor settings [3,9,10]. Monotonous diets are closely associated with food insecurity [9], resulting in malnutrition.

Promoting dietary diversity has been suggested as one of the strategies to alleviate nutritional problems that occur due to inadequate intake of micronutrients and food insecurity [9]. The consumption of a wide variety of foods among and within food groups helps in ensuring adequate intake of micronutrients which are essential to nutritional adequacy [9]. According to the Food and Agriculture Organisation (FAO) [11], dietary diversity is a qualitative measure of food consumption that reflects household access to a variety of foods, and is also a proxy for nutrient adequacy of the diet of individuals. Dietary diversity can be assessed by using tools such as dietary scores which sum the number of food groups consumed over a reference period and these are good proxies of overall dietary quality [11,12,13] and are useful indicators of household food security [14].

A lack of dietary diversity is a severe problem globally [9], which is mostly experienced by the poorest households. Women of reproductive age are at high risk of micronutrient deficiencies [4,15], yet information on micronutrient deficiencies and dietary patterns among women is scarce. Studies have shown that women are more vulnerable and in many cases, there is uneven control of resources in the households which makes the experience of food insecurity to be gender biased [16,17]. Sasson [18] reported serious harmful situations faced by women and female teenagers who receive less food than their male counterparts in the same households during times of food insecurity. For women of reproductive age, studies have shown that consuming micronutrient-poor diets may harm both women and their infants [19,20]. Studies have also shown that when households experience food insecurity, many mothers reduce their own intakes to secure those of infants and small children to avoid child malnutrition [21,22], and some women may rely on less expensive foods that are energy rich but nutrient poor [23]. That is, the diets often contain little or no fresh vegetables and fruit, and are low in animal source foods rich in iron and Vitamin A [10], or the diets would be rich in processed foods that are high in sugar and fat [24]. Laraia et al. [25] also reported that household food insecurity is associated with reduced micronutrient intake among women of reproductive age due to a decrease in fruit and vegetable consumption. Although South Africa may be food secure at a national level, large numbers of households within the country are food insecure [26,27]. Many households in the country are living “close to the edge”, below the food poverty line of R321 per month, and therefore, they consume poor quality diets and alter their consumption routines to fit with their poverty [28]. South Africa is listed by the World Health Organisation (WHO) as one of 36 countries with the highest burden of undernutrition [26,27], yet little is known about the dietary diversity of women despite the significant recognition it has in health and nutrition. Stunted growth and underweight are major nutritional disorders in the country [29,30] because many poor people in the country tend to cope with poverty by adopting monotonous diets as well as processed foods higher in saturated fat, sugar, and salt [24,31,32]. Poor households are mostly affected and this is exacerbated by rising food prices, widespread chronic poverty, and unemployment [26,33], which is also weakening the informal safety nets, especially in urban areas [32,33]. This increases the proportions of households vulnerable to food and nutrition insecurity as hunger and malnutrition continue to increase in the country.

In South Africa, two-thirds of the population now live in urban areas [28] and the majority of the population is in the process of transition from rural to urban lifestyles. The majority of the population is accessing food commercially through markets due to a decline in smallholder agriculture [34,35]. Hunger and nutrient deficiencies are now common across South Africa, in urban, peri-urban informal settlements, and rural areas [36,37,38]. For example, Shisana et al. [38], found that only 45.6% of the South African households were food secure and household dietary diversity was low (Dietary Diversity Scores (DDS) = 4.2) with 40% of the households having scores lower than four. However, the available national dietary data for South Africa focused at household level. Little is known about the nature and relative magnitude of individual dietary diversity, particularly women of reproductive age (WRA) along the agro-ecological zones (AEZs) and the rural–urban continuum of small- and medium-sized towns yet that is where the bulk of urbanisation is happening. The limited information regarding the changes in household and individual food security and nutrition along agro-ecological gradients as well as along the rural-urban gradient provides the foundation for this research.

The aims of the study were to determine the dietary diversity of women in three mid-sized towns in South Africa using the MDD-W indicator. The study also aimed to compare the MDD-W along an agro-ecological gradient and the rural-urban (R-U) gradient in each town and explore the relationships between MDD-W and household characteristics such as wealth, household size, availability of land, and food expenditure. We hypothesised that:

1. Minimum Dietary Diversity for WRA (MDD-W) decreases with a decrease in agro-ecological potential.
2. Peri-urban women would report higher levels of food insecurity than those in more rural and urbanised areas.
3. Monotonous diets would be observed for women from poor households and these will be based mainly on starchy staples, with little vegetables and fruit.
4. The MDD-W and food security would be associated with household size, wealth, and food expenditure.

## 2. Materials and Methods

### 2.1. Study Sites

**Figure 1:**
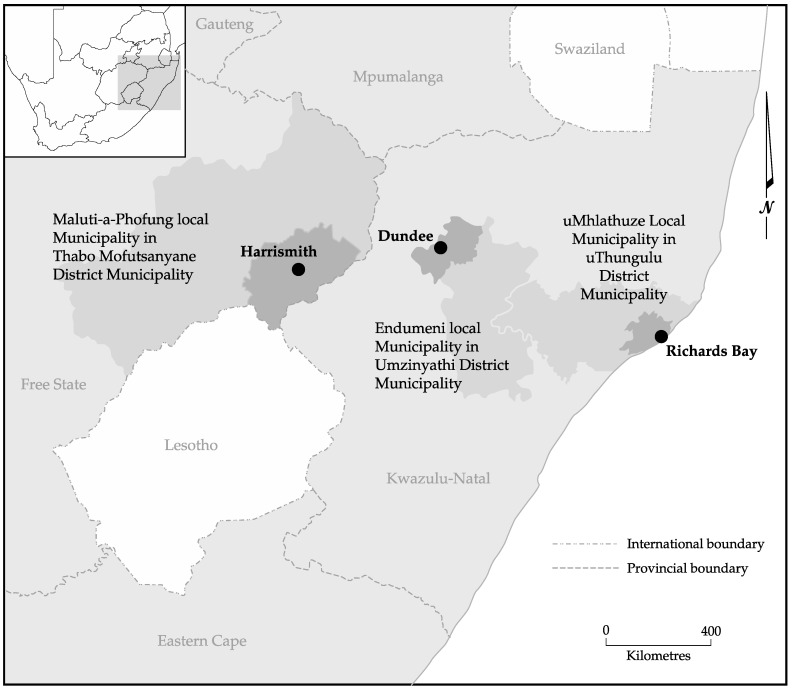
Location of study towns in South Africa.

The study considered the agro-ecological zones (AEZs), which are geographical areas exhibiting similar climatic conditions that determine their ability to support rain-fed agriculture. These are influenced by latitude, elevation, and temperature, as well as seasonality, and rainfall amounts and distribution during the growing season [39]. The study was carried out in three medium-sized towns in South Africa, namely Richards Bay, Dundee, and Harrismith (Figure 1). The towns were selected according to their position along an agro-ecological gradient, with Richards Bay being a coastal and relatively warm and wet town (approximately 970 mm per annum), while Harrismith is an inland and dry town (approximately 622 mm per annum), and Dundee being intermediate (inland and 683 mm per annum). The seasonality of the rainfall increases along this gradient, along with the severity of winter temperatures. Thus, the gradient also reflects one of declining suitability for rain-fed agriculture, from high in Richards Bay to low in Harrismith, where rural farms mostly practice cattle ranching. Each study site consisted of the rural, peri-urban, and urban complex and data was collected along the rural–urban continuum.

### 2.2. Sampling

All interviews were conducted in the respondent’s preferred language of isiZulu in Richards Bay and Dundee, and Sesotho in Harrismith or English. Enumerators were trained on how to conduct interviews using the questionnaire so as to provide full understanding of the administered questions. Ethics approval was granted by the Rhodes University Ethical Standards Committee with permit number RU-HSD-14-08-0012.

Dietary diversity data were obtained through administering questionnaires to randomly selected households at each site. Within each town, 200 households were randomly selected, comprising of 60 rural households, 80 peri-urban households, and 60 urban households. Random sampling using ArcGIS software was used and GPS coordinates were generated for each selected household. In the case of a woman from the selected households refusing to participate or no women of reproductive age being available for interview, then the nearest house to the left was interviewed. However, not all selected households agreed to participate in the interviews. This left a total of 183 individuals interviewed in Richards Bay, 173 in Dundee, and 198 in Harrismith.

Information on household characteristics such as the household size, age, gender of household head, sources of food, income, land acquisition, wealth (assets acquired by household), and food expenditure per week were also asked. An index of wealth was created by combining information on the household’s possessions which included car/truck, motorbike, tractor, bicycle, fridge, television, radio, cattle/goats, chickens, cell phone, house, and electricity. For each household, the number of each asset was normalized (by dividing with the highest number obtained in each category for all households) then all summed to get a wealth index per household, which could range from zero to 12.

### 2.3. Minimum Dietary Diversity for WRA (MDD-W)

Women’s dietary diversity can be measured using the minimum dietary diversity for WRA (MDD-W) indicator which aims to reflect individual dietary intake and nutrient adequacy of women of reproductive age [12,13]. Minimum dietary diversity for WRA, which is the sum of food groups consumed by women over a reference period, can also be used as a measure of household access to a micronutrient rich diet [10,11]. Therefore, MDD-W is noted as a conservative estimate of household nutritional security as well as micronutrient adequacy of the women’s diet [10,11,12,13]. MDD-W is associated with household food production [4], wealth [4,40], knowledge of nutritional requirements, and household size [4].

Dietary diversity was determined using the standard 48 h recall technique adopted from FAO [13] method. The MDD-W approach assumes that the respondent would know all the meals she prepared, served, and consumed. Information on the food that was consumed by the women was collected from a woman of reproductive age (15–49 years old) and ideally the person in the household who prepares most of the meals. If the person who did most of the cooking was a stay-out employee (domestic worker), she was required to report on the meals she had cooked for the household and not what she consumed herself. The women were asked to recall and name all the food they had consumed for the past two days (day and night), that is, all dishes, snacks, and drinks. They were encouraged to remember all the food consumed per meal and in-between meals. The women were also asked to fully describe all the ingredients in mixed dishes, the source of ingredients (bought, grown, collected, or donated), and source of energy used to prepare the food. All the ingredients were coded into a list of 14 major food groups which are aggregated to ten for analysis [13]. Each household was visited twice during the pre-harvest period (summer) between October and November 2014 and the survey was repeated again in June 2015 during the post-harvest period (winter). The same individual was interviewed during all the visits. Two seasons covered possible variations in seasonal local diets, nutrition, and food access.

The information obtained was used to calculate the MDD-W for each woman following FAO and FHI 360 [13]. Following FAO and FHI 360 [13], MDD-W is defined as the sum of food groups consumed by a woman from the total of ten food groups required. The ten food groups included: (1) Grains, white roots and tubers, and plantains (also known as starchy staples); (2) Pulses (beans, peas, lentils); (3) Nuts and seeds; (4) Dairy; (5) Meat, poultry and fish; (6) Eggs; (7) Dark green leafy vegetables; (8) Other Vitamin A-rich fruits and vegetables; (9) Other fruits and (10) Other vegetables. The food groups which are included in the MDD-W mostly reflect the diet quality with the probability of minimum micronutrient adequacy of the women’s diets summarized across 11 important micronutrients which are vitamin A, thiamine, riboflavin, niacin, vitamin B6, folate, vitamin B12, vitamin C, calcium, iron, and zinc [12]. The fats and oils food group was not included for MDD-W because it does not contribute to the micronutrient density of the diet [11]. However, as recommended by FAO [11], the proportion of individuals consuming fats and oils could be calculated as a separate indicator since oil improves the absorption of plant scarotenoids and fat-soluble vitamins and is an important contributor to energy density. Using MDD-W allows grouping women into classes of food secure or insecure. A woman was classified as having poor dietary diversity and food insecure if she had consumed <5 food groups or had achieved MDD-W with good dietary diversity and was food secure if she had consumed ≥5 food groups in the previous 48 h [13].

### 2.4. Statistical Analysis

Data were entered and cleaned using Microsoft Excel and all statistical analyses were performed using Statistica version 12 (StatSoft Inc., Tulsa, OK, USA). Descriptive data are presented as means and standard deviations (mean ± SD). Data on MDD-W for each town were not normally distributed (tested via Kolmogorov–Smirnov test), therefore comparisons between MDD-W of first and second visit in summer and again between winter and summer were done using a non-parametric Mann Whitney U test. Since there was no significant difference between the medians, data were treated as one data set and was used in all analyses. The differences MDD-W between towns and locations were tested using a 2-way ANOVA and a post-hoc analysis was performed to provide specific information on which differences were significant. As the data were not normally distributed, the Spearman correlation test was used to examine MDD-W as the response variable as a function of wealth, household size, and food expenditure. Thus, an analysis was done to determine if there were any associations between MDD-W as a measure of food security and wealth, household size, and food expenditure. Statistical significance was set at *p* < 0.05 for all tests. The South African Rand to US dollar exchange rate was approximately 11:1 at the time.

## 3. Results

### 3.1. Household Characteristics

The sample consisted of 554 women of reproductive age (WRA) aged between 15–49 years with a mean age in the three towns ranging from 29 ± 9.0 to 33 ± 10.8 years (Table 1). The household size for the study sites ranged from 6 ± 2.2 to 8 ± 4.2 persons and the majority of the households were female-headed. More than 80% of households in Dundee and Harrismith received some form of cash income whilst only 59% of household in Richards Bay did so. Households in Richards Bay were spending less cash per week on purchasing food than in Dundee and Harrismith. The wealth index was similar for all towns although it was greatest in Richards Bay, with Dundee having the smallest. A greater percentage of households in Richards Bay had land available for own production whilst only 1 in 2 households in Dundee and about 1 in 4 households in Harrismith did so.

**Figure 2:**
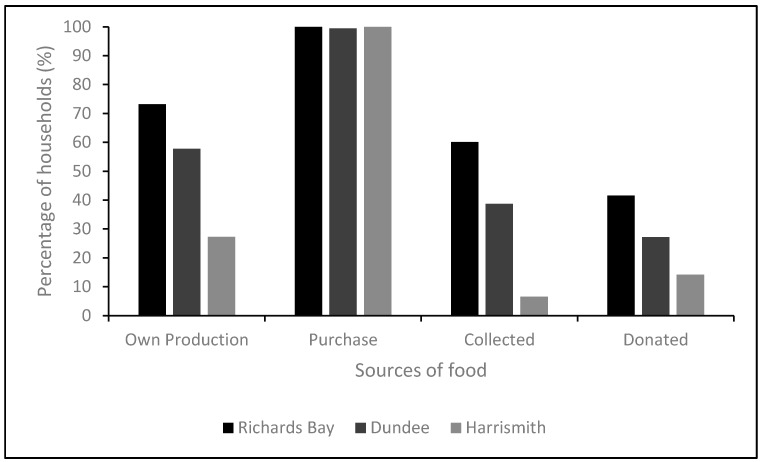
Percentage of women obtaining food from different sources.

In general, although the greatest part of the food consumed by households in the three towns was purchased, more than 70% of households in Richards Bay produced some of their own food, 66% collected some food from the wild, and about 40% received some food donations. This was different from Dundee and Harrismith, where less than 55% of households in Dundee and less than 30% in Harrismith obtained food from other sources (Figure 2). The general food acquisition by the households in all sites followed a similar pattern along the agro-ecological gradient. All households purchased food from different sources in all three sites. However, there is a clear decrease in own production and self-collection with a decrease in agro-ecological gradient from Richards Bay to Harrismith. Donations of food to households in the three towns also followed a similar pattern (Figure 2).

**Figure 3:**
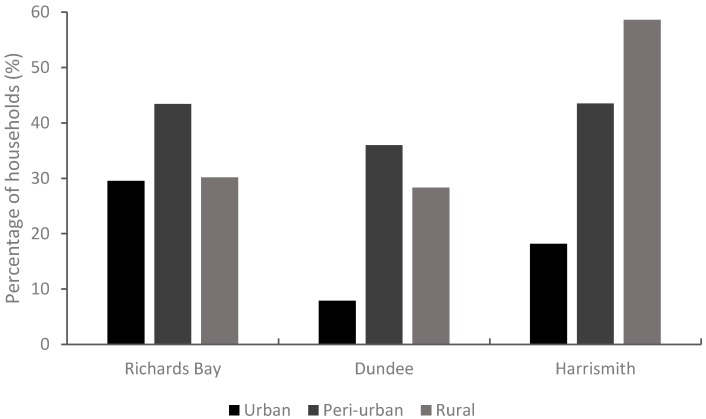
Percentage of households living in extreme poverty, i.e., below the South African food poverty line (FPL) of R321 per capita per month as per March 2011 [28].

When using the food poverty line (FPL) (which is the level of consumption below which individuals are unable to purchase sufficient food to provide them with an adequate diet (Stats SA, Pretoria, South Africa, 2014)) to define households living in extreme poverty, about 36% of households in Richards Bay were living in extreme poverty, 27% in Dundee, and 41% in Harrismith. Along the rural–urban continuum, levels of extreme poverty were lowest in urban areas at all sites and highest in the peri-urban and rural locations. In Richards Bay and Dundee, the highest percentage of households living in extreme poverty was in the peri-urban zone, whilst in Harrismith it was in the rural location (Figure 3).

### 3.2. Dietary Diversity

**Figure 4:**
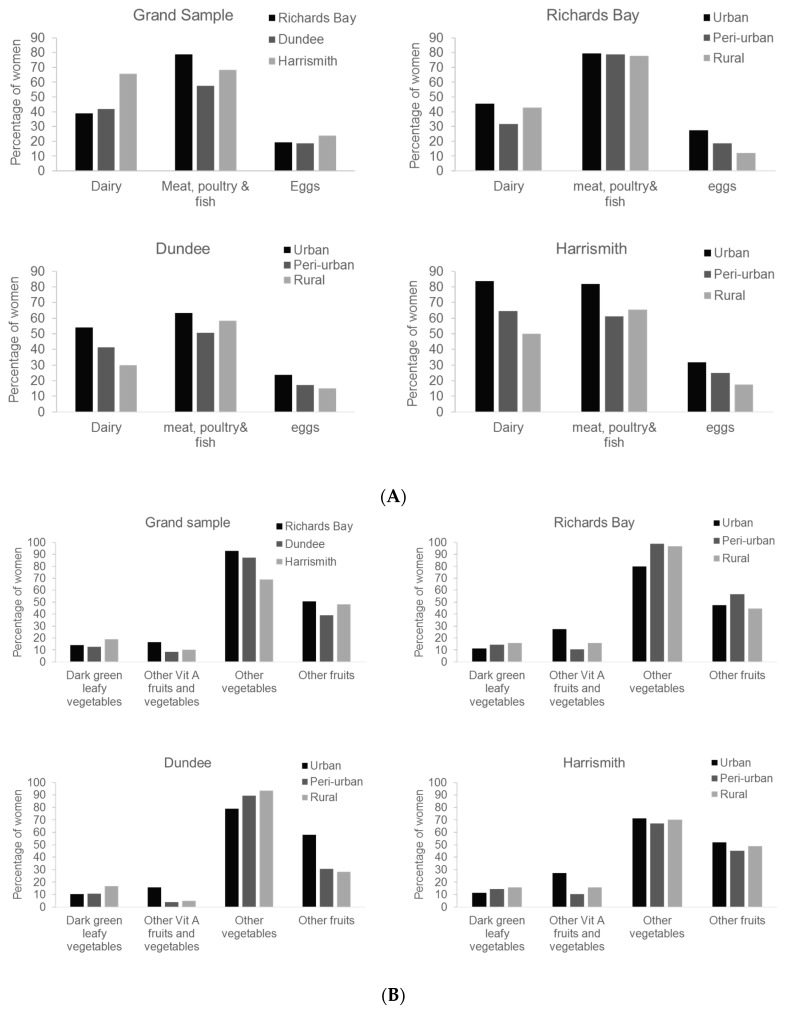

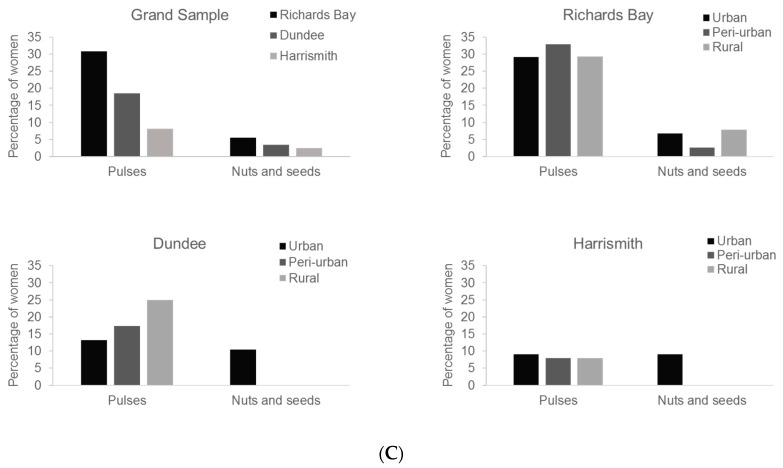
(**A**–**C**) Percentage of women consuming nutrient-rich foods in the previous 48 h along the agro-ecological gradient and rural-urban continuum. (**A**) Animal-source foods; (**B**) Fruits and vegetables; (**C**) Pulses, nuts, and seeds.

The food group that was consumed by 100% of women in all towns was the grains, white roots and tubers, and plantains group, which is sometimes called the starchy staples group. The foods in this group which were consumed by women were mostly pap (cooked maize-meal), samp (crushed maize), rice, potatoes, amadumbe (*Colocasia esculenta*), bread, and pasta. The food groups consumed by at least 50% of women in all towns were the meat, poultry, and fish group and other vegetables (mostly cabbage and onion) (Figure 4A,B, respectively). The greatest percentage of women consuming these food groups was in Richards Bay. The dairy group and other fruits group were consumed by greater than 50% women in Harrismith and Richards Bay, respectively (Figure 4A,B). The consumption of the dairy products followed the rural–urban continuum in all towns, with more urban women consuming foods from this group than peri-urban and rural women. The consumption of pulses, nuts, and seeds (Figure 4C) and other Vitamin A fruits and vegetables (Figure 4B) followed the agro-ecological gradient with Richards Bay having the greatest prevalence and Harrismith the least, although these food groups were consumed by less than 35% of women in all towns and locations. In Dundee and Harrismith, nuts and seeds were only consumed by women living in urban areas. Generally, in all towns, dark green leafy vegetables and vitamin A-rich vegetables and fruit were not widely consumed (<30% of women). Vitamin A-rich vegetables and fruit were eaten more in Richards Bay than in Dundee and Harrismith, and dark green leafy vegetables were consumed more in Harrismith than the other two. Eggs were infrequently eaten in all towns and they were mostly consumed in urban areas, followed by peri-urban and lastly in rural areas (Figure 4A). Women’s diets along the rural-urban continuum revealed a clear pattern of highest minimum dietary diversity within the urban and lowest in either the rural or peri-urban locations (Figure 4).

### 3.3. Differences in MDD-W between and within Towns

The mean MDD-W for the sample was 3.46 ± 0.99. The percentage of WRA who achieved minimum dietary diversity was 25%, and they are more likely to have higher (more adequate) micronutrient intakes than the 75% of women who did not meet the minimum dietary diversity (Table 2). Overall, mean MDD-W for the towns was generally low. There was a significant difference in MDD-W along the agro-ecological gradient (*F*_2, 549_ = 16.04, *p* = 0.00001), with Richards Bay being higher than the other two, which were not different from one another.

Within sites, mean MDD-W was higher in the urban location and lowest in the rural location, with the peri-urban location being intermediate (Table 2). There was a significant difference in MDD-W along the rural–urban continuum (*F*_2, 549_ = 10.96, *p* = 0.00002), being higher in the urban than the other two which were not significantly different. In all sites, highest MDD-W were observed in the urban location and the lowest MDD-W were observed in the rural locations in Richards Bay and Harrismith, but in peri-urban for Dundee. The MDD-W were mostly lower than five food groups consumed by women in all towns.

### 3.4. Association of MDD-W with Socio-Economic Indicators

Significant positive correlations were found between the MDD-W and food expenditure and wealth indices in all the towns. The strongest correlations were found for Harrismith (Table 3). In Richards Bay, a significant positive correlation was also found between MDD-W and access to land (Table 3). In all three towns, there was no significant correlation between MDD-W and household size.

Within sites, the strongest significant positive correlations were found between the MDD-W and food expenditure and wealth indices in peri-urban locations of Richards Bay and Harrismith, and in Harrismith’s rural location (Table 4). Significant correlations were also found between MDD-W and food expenditure in urban areas of Richards Bay and Dundee as well as between MDD-W and wealth in rural Dundee. There were no significant correlations between MDD-W and food expenditure and wealth in the Richards Bay rural, Dundee peri-urban, and Harrismith urban areas.

## 4. Discussion

The results of this study form the basis of a deeper understanding of the dynamics of food security and nutrition in a particular context along agro-ecological gradients and along the rural-urban gradients in medium-sized South African towns. The MDD-W tool has been used as an indicator of access to food and was able to distinguish women with different levels of vulnerability to food insecurity through identifying those with poor access to food. The diets of most women living in the study sites were dominated by starchy staples, predominantly maize meal, which provide energy, varying amounts of micronutrients (e.g., certain B vitamins provided by grains), and varying amounts of anti-nutrients [13]. These findings are consistent with those reported by Arimond et al. [10], Faber et al. [24], Schönfeldt et al. [31], Oldewage-Theron and Kruger [41], and Acham et al. [42], who reported that cereals and starchy foods, especially maize based foods, are the most consumed foods by South Africans. Although women in all towns frequently consumed other vegetables (which consisted predominantly of cabbage and onion) and meat, poultry, and fish, there was very limited intake of fruit, including other Vitamin A-rich fruits and vegetables as well as dark green leafy vegetables. This is consistent with Arimond et al. [10], Faber et al. [24], and Schönfeldt et al. [31], who found that most poor communities are at high risk of inadequate micronutrient intake due to low intake of fruits and vegetables. Women in the study sites had limited intake of micronutrients, as well as of other phytochemicals and fiber which are obtained from consuming different fruits and vegetable groups. Pulses, nuts, and seeds which are rich in vegetable protein, B vitamins, unsaturated fatty acids, fiber, and minerals which have unique health benefits were rarely consumed.

The MDD-W were mostly lower than five food groups consumed by women in all towns and locations. When using a mean MDD-W of five or less food groups to define a poor dietary intake and food insecurity, results revealed that 75% of women in the study sites had failed to achieve the minimum dietary diversity, and are hence more likely to have inadequate micronutrient intake which increases their vulnerability to food insecurity. Only one in four women were food secure and had a good quality diet. This is within the range reported in other South African studies where very low dietary diversity scores were observed [38,41,43], although there are no reports on women of reproductive age. Consuming a more diverse diet is beyond the reach of most people in South Africa because of high poverty levels due to high unemployment, increasing food prices, and abandonment of agriculture which leads to over-dependence on purchasing food from markets. It has been reported that the increase in food prices, especially cereals, has an impact on food consumption among vulnerable households. In this case, most households switch to cheaper and less nutritious foods that satisfy hunger, but compromise the quality of the food consumed [44]. Households may also decrease dietary diversity in response by reducing portion sizes and frequency of meals [44,45]. This was confirmed in the present study by strong positive correlations between wealth, food expenditure, and MDD-W. In South Africa, the majority of households are struggling to obtain a decent income [43] and low-income households cannot afford a diverse diet, especially vegetables [46]. Women of reproductive age are at great risk of low dietary diversity in these vulnerable households [10], since women may find it difficult to purchase enough food to feed the entire household [43] and may prioritize their children’s food consumption over their own [21,22].

The results from the present study showed that women residing in coastal areas with wetter climatic conditions and longer growing seasons that sustain viable rain-fed agriculture were more food secure compared to those living in inland areas with drier climatic conditions where households mostly rely on purchasing food. Women in Richards Bay had the highest mean MDD-W followed by those residing in Harrismith, whereas women in Dundee had the lowest values. Higher MDD-W in Richards Bay could be attributed to wetter and warmer climatic conditions and a longer growing season which favor agriculture, compared to Dundee and Harrismith where drier and cool conditions make agricultural production less viable. Dietary diversity was strongly correlated with access to use of land in Richards Bay, reflecting the greater engagement in own production in this town which suggests that own production improves the quality of women’s diets at this site. For example, farming has been shown to increase food security for low-income households, and farm produce has been marked as an alternative to imported foodstuffs which is cost effective for poor households [47,48]. Subsistence agriculture in rural Richards Bay produces high quality food and additional income from selling of surplus produce, thus enabling some households to acquire other essential products from the supermarkets. There is lower dependence on food purchasing in Richards Bay compared to Dundee and Harrismith, where the majority of households are not producing their own food and are net buyers of food. Most women in Dundee and Harrismith are food insecure and this has a negative impact on a large proportion of households already vulnerable to food insecurity, thereby increasing hunger and malnutrition. A decline in smallholder agriculture in South Africa has exacerbated the levels of food insecurity as the majority of the population is accessing food commercially through markets [34,35,37], therefore households limit both the quality and quantity of their dietary intake. Due to increased urbanization, household agriculture is becoming less significant as a primary food source, yet food prices are rising faster than inflation, hence increasing food insecurity for the poor households [36] who cannot afford to purchase diverse foods to achieve quality dietary intake rich in micronutrients. Therefore, farming or gardening has the potential to improve the food security of poor households in both rural and urban areas by increasing food supply and dietary intake as food prices increase [49,50].

Accessing food through collecting from open spaces rather than purchasing has also been noted to have increased MDD-W in Richards Bay. Almost two-thirds of households in Richards Bay obtained some of their food, especially vegetables and fish, from the wild, a proportion which is considerably higher than households in Dundee and Harrismith. Wild foods can support households that are or have experienced a shock and are important contributors to food security. For example, Mojeremane and Tshwenyane [51] noted that wild fruit were consumed as a form of reducing the risks of food insecurity in Botswana. Jumbe et al. [52] also noted that wild foods such as wild fruit, wild leafy vegetables, wild mushrooms, tubers, edible insects, and honey from the miombo woodlands improved food and nutrition security for most rural communities in Zambia through increase in dietary diversity as most are rich in micronutrients. Edible insects and fish are rich in protein, while wild fruits and wild vegetables are good sources of Vitamin A and fiber which are some of the 11 micronutrients expected to be consumed by women to achieve MDD-W. Studies by Arnold et al. [53] and Legwaila et al. [54] also reported that wild foods, especially indigenous fruits and leafy vegetables, provide an important source of food that are high in micronutrients and supplement diets thereby improving dietary diversity.

Comparisons along the rural–urban continuum showed that levels of food insecurity were lower in urban areas, and fruit (Vitamin A-rich and other fruits) were mostly consumed with women with high MDD-W in urban locations. This study indicated that the urban women are better off than their peri-urban and rural counterparts as food was readily available in urban areas and the only limitation was physical and financial access. The study has shown the strong positive correlations between wealth, food expenditure, and MDD-W in both peri-urban and rural locations in the study sites, thus increasing the number of households living in extreme poverty. It has been noted by Jacobs [55] and Rudolph et al. [56] that poor households are more likely to suffer from food shortages, hence low dietary intake than wealthier households since food expenditure makes up a large share of their spending, thereby causing them to be more vulnerable to the impacts of rising food prices. Although households in the rural areas may grow some of their food, most rely on food purchasing and women in these areas had limited access to affordable food and were facing higher prices for food as they had to travel to distant markets (usually to town) to buy food. The peri-urban populations were more food insecure because of high levels of poverty as a larger percentage of women in peri-urban locations were living in extreme poverty, below the FPL. Households living below the FPL are said to consume poor-quality diets and alter their consumption routines to fit with their poverty [28] which is also evident in this study, as shown by the lowest MDD-W in peri-urban locations. Also, peri-urban households have limited access/entitlements to land (as most occupy land illegally), which can also make them more vulnerable to food insecurity than both their urban and rural counterparts who have access/entitlement. Peri-urban dwellers are therefore more sensitive to changes in incomes and food prices than the rural and urban populations because they have limited safety nets to absorb income or price shocks as they purchase more, rather than growing their own food. Naicker et al. [57] also noted that factors such as low income, low asset ownership, and unemployment increased the risk of food insecurity in households in informal settlements in Johannesburg. Hence, poor socio-economic status has an impact on household MDD-W and food insecurity to a larger extent in peri-urban locations, as was also found by Labadarios et al. [43] and Oldewage-Theron et al. [45] in South Africa. The peri-urban households are more food insecure than rural and urban households, although the substantial increases in the dependence on market purchases is affecting both urban and rural households [49].

## 5. Conclusions

Findings from this study emphasised that increased dependence on purchasing food, decreased own production, poor climatic conditions, lack of suitable land, limited access to food due to lack of income, and high food prices are the main causes of food insecurity and low dietary diversity in women living in mid-sized towns in South Africa, especially those residing in peri-urban and rural locations. Therefore, food security intervention programmes should focus on developing both the rural and peri-urban communities. There is need to improve diets and dietary diversity for women of reproductive age in South Africa by practising own production through home gardens or engaging in community gardening. Household food production has been noted in this study as the most important way to improve food security and dietary diversity of women from poor households through increased food access, consumption of fresh nutritious food, and reduced dependence on market purchase. Studies have shown that community gardening projects can reduce food insecurity and improve dietary (micro-nutrient) intake through consumption of fresh vegetables [58]. Furthermore, home gardens have direct contributions to household food security through increasing availability, accessibility, and utilization of food, which are the three pillars of food security. The observed relationship between access to land and dietary diversity along the agro-ecological gradient has an important implication for municipalities making available such land, along with relevant support and inputs to affected women so that they diversify food access through production of their own food. In areas like Dundee and Harrismith which are often affected by droughts, research should focus on climate-smart agriculture approaches as well as use of drought resistant crops. Furthermore, as food prices are continuing to increase gradually and household food insecurity becomes worse, ways to shift the ‘income circumstances’ of these poor households needs to be advocated. Therefore, focus should be placed on capacity building and employment creation which may improve the poverty status of women in the study sites.

## Figures and Tables

**Table 1:** Comparison of household characteristics in study sites.

Variable	Richards Bay (*n* = 183)	Dundee (*n* = 173)	Harrismith (*n* = 198)	All (*n* = 554)
Respondent age (mean ± SD) (year)	29 ± 9.0	33 ± 10.8	33 ± 9.9	32 ± 10
Household size (mean ± SD) (number of people)	7 ± 4.6	8 ± 4.2	6 ± 2.2	7 ± 4
Household head (%)				
*Male*	42	36	47	42
*Female*	58	64	53	58
Some form of cash income (%)				
*None*	41	20	9	23
*One income*	49	59	72	60
*Two or more incomes*	10	21	19	17
Food expenditure (mean ± SD) (Rand/week)	196 ± 180	333 ± 253	323 ± 271	284 ± 246
Wealth index (see methods)	2.6 ± 0.6	2.3 ± 1.0	2.5 ± 0.9	2.5 ± 0.8
Households with land for own production (%)	73	57	27	52

**Table 2:** Individual dietary diversity scores (MDD-W) of women for three sites and three locations per site. (Unlike superscripts indicate significant differences).

Town	MDD-W (Mean ± sd)	Location	MDD-W (Mean ± sd)	Percentage of Women	
				<5 Food Groups	≥5 Food Groups
**Richards Bay** **(*n* = 183)**	3.78 ± 0.07 ^a^			66	34
		Urban (*n* = 44)	4.79 ± 0.15 ^a^	68	32
		Peri-Urban (*n* = 76)	3.19 ± 0.11 ^a^	80	20
		Rural (*n* = 63)	3.40 ± 0.13 ^a^	78	22
**Dundee** **(*n* = 173)**	3.21 ± 0.08 ^b^			87	13
		Urban (*n* = 38)	3.74 ± 0.16 ^a^	74	26
		Peri-Urban (*n* = 75)	3.09 ± 0.11 ^b^	91	9
		Rural (*n* = 60)	3.03 ± 0.12 ^a/b^	93	7
**Harrismith** **(*n* = 198)**	3.36 ± 0.07 ^b^			73	27
		Urban (*n* = 55)	4.05 ± 0.12 ^a^	67	33
		Peri-Urban (*n* = 85)	3.78 ± 0.10 ^b^	73	27
		Rural (*n* = 58)	3.53 ± 0.12 ^b^	78	22
**All towns** **(*n* = 554)**	3.46 ± 0.99			75	25
		Urban (*n* = 137)	3.82 ± 0.09 ^a^	69	31
		Peri-urban (*n* = 236)	3.37 ± 0.07 ^b^	81	19
		Rural (*n* = 181)	3.31 ± 0.07 ^b^	83	17

**Table 3:** Spearman correlations between MDD-W and selected socioeconomic indicators in the three towns.

Town	Household Size	Food Expenditure per Week	Wealth Index	Access to Land
**Richards Bay**	−0.017	0.294 ^a^	0.333 ^a^	0.155 ^b^
**Dundee**	−0.063	0.286 ^a^	0.267 ^a^	0.117
**Harrismith**	−0.101	0.533 ^a^	0.341 ^a^	0.031

Correlations are significant at *p* < 0.05 (^a^) and *p* < 0.005 (^b^).

**Table 4:** Spearman correlations between MDD-W and both wealth index and food expenditure within towns.

Town	Location	Food Expenditure per Week	Wealth Index
**Richards Bay**	Urban	0.350 ^a^	0.251
	Peri-urban	0.467 ^b^	0.429 ^b^
	Rural	−0.009	0.236
**Dundee**	Urban	0.427 ^b^	0.156
	Peri-urban	0.127	0.132
	Rural	0.243	0.338 ^b^
**Harrismith**	Urban	0.099	−0.011
	Peri-urban	0.549 ^c^	0.295 ^b^
	Rural	0.468 ^c^	0.422 ^b^

Correlations are significant at *p* < 0.05 (^a^), *p* < 0.001 (^b^) and *p* < 0.0001(^c^).
